# The Combination of Catechin and Epicatechin Gallate from Fructus Crataegi Potentiates β-Lactam Antibiotics Against *Methicillin-Resistant Staphylococcus aureus* (MRSA) *in Vitro* and *in Vivo*

**DOI:** 10.3390/ijms14011802

**Published:** 2013-01-16

**Authors:** Rongxin Qin, Kangkang Xiao, Bin Li, Weiwei Jiang, Wei Peng, Jiang Zheng, Hong Zhou

**Affiliations:** 1 Department of Pharmacology, College of Pharmacy, The Third Military Medical University, Chongqing 400038, China; E-Mails: michel_0415@163.com (R.Q.); xiaomick1985710@126.com (K.X.); libin6033@sina.com (B.L.); jww613@sina.com.cn (W.J.); pengwei002@126.com (W.P.); 2 Medical Research Center, Southwestern Hospital, The Third Military Medical University, Chongqing 400038, China

**Keywords:** fructus crataegi, (+)-catechin, (−)-epicatechin gallate, synergistic effect, MRSA, drug accumulation

## Abstract

Fructus crataegi (hawthorn) is the common name of all plant species in the genus *Crataegus* of the Rosaceae family. In the present study, three monomers of (+)-catechin (C), (−)-epicatechin gallate (ECg) and (−)-epigallocatechin (EGC) were isolated from the hawthorn under the guide of antibacterial sensitization activity. The bioactivity of the composite fraction in enhancing the antibacterial effect of oxacillin against methicillin-resistant *Staphylococcus aureus* (MRSA) was greater than that of the individual monomer of the hawthorn extract *in vitro*. Two-fold dilution and checkerboard methods were used to analyze antibacterial activity and screen for the combination and proportion of monomers with the best bioactivity. The result showed that C (128 mg/L) combined with ECg (16 mg/L) had the greatest effect and the combination also reduced the bacterial load in blood of septic mice challenged with a sublethal dose of MRSA, increased daunomycin accumulation within MRSA and down-regulated the mRNA expression of *norA*, *norC* and *abcA*, three important efflux pumps of MRSA. In summary, C and ECg enhanced the antibacterial effect of β-lactam antibiotics against MRSA *in vitro* and *in vivo*, which might be related to the increased accumulation of antibiotics within MRSA via suppression of important efflux pumps’ gene expression.

## 1. Introduction

Methicillin-Resistant *Staphylococcus aureus* (MRSA) is the major multi-resistant pathogen causing serious healthcare-associated and community-onset infection, which carry high morbidity and mortality [1]. In fact, MRSA bacteria are resistant to nearly all types of antibiotics, especially β-lactam antibiotics [2].

The main resistance mechanisms of MRSA to the β-lactam antibiotics are a large expression of β-lactamase to destroy β-lactams by hydrolysis, an acquisition of the *mecA* gene to encode the penicillin-binding protein 2a (PBP2a) with low affinity to β-lactam antibiotics [3], and an expression of efflux pumps to extrude antibiotics or other toxic agents from the pathogen [4]. To date, more than 10 efflux pumps have been discovered for *S. aureus* [5]. Although these pumps show different substrate specificity, most of them are capable of extruding compounds of different kinds of antibiotics, thus providing the pathogen the means to develop a multidrug resistance phenotype.

There are usually two common strategies to overcome MRSA resistance. One approach is to develop direct antimicrobial agents. However, researching new agents is very difficult, and the propensity to increase resistant strains will occur when the agent applied is extensively used in clinic. Another approach is to develop new agents that can enhance the effect of existing antimicrobial drugs—these are called antibacterial drug sensitizers—via modifying the bacterial phenotype to sensitize MRSA to previously ineffective antibiotics rather than the direct killing of bacteria. The advantage of the agents is that there is little or no direct selective pressure, and thus resistant strains are less likely to emerge [6]. Therefore, the antibacterial drug sensitizers have increasingly gained attention.

Natural products are valuable sources of antibacterial drug sensitizers. Some natural products can enhance the efficacy of β-lactam antibiotics, and therefore they are termed intensifiers of β-lactam susceptibility in MRSA (ILSMRs) and this effect is known as the ILSMR effect [7]. Baicalin [8], diterpenes [9], tellimagrandin I [10], corilagin [11], tripeptide [12], epigallocatechin gallate (EGCg) [13] and epicatechin gallate (ECg) [14,15] are previously reported ILSMRs. Among them, EGCg and ECg belong to same category of compounds as catechin, which is polyphenol present in many plants such as *Camellia sinensis* (green tea) and *Acacia catechu* (L. f.) Willd. (catechu). Catechin can intensify the susceptibility of MRSA to β-lactam antibiotics despite themselves having very weak or no antimicrobial effects against MRSA [16].

Fructus crataegi (hawthorn) is the common name of all plant species in the genus *Crataegus* of the Rosaceae family. It is widely used as a traditional medicinal plant in some countries. The fruits of native hawthorns are also edible. The extracts and compounds from hawthorn have been reported to show a variety of pharmacological activities, such as a protective effect on patients with heart failure [17], as well as an increased force of myocardial contraction [18], improved coronary circulation [19], antioxidant effects [20] and antimicrobial activities [21].

Previously, the crude extract of hawthorn was found to have ILSMR effect in our lab. In the present study, three monomers of (+)-catechin (C), (−)-epicatechin gallate (ECg) and (−)-epigallocatechin (EGC) were isolated from hawthorn. Because the bioactivity of the composite fraction in enhancing the antibacterial effect of oxacillin against MRSA was greater than that of the individual monomer *in vitro*, the two-fold dilution and checkerboard methods were used to analyze antibacterial activity and screen for the combination and proportion of monomers with the greatest intensifying of β-lactam susceptibility against MRSA. Subsequently, the effects of combinations with different antibiotics against standard or clinical MRSA strains were investigated *in vitro* and *in vivo*. Lastly, the possible mechanisms behind them were also investigated.

## 2. Results and Discussion

### 2.1. Combination of C and ECg Produces the Greatest ILSMR Effect in a Screening of Constituents from a Bioactive Fraction of Hawthorn

In the present work, C, ECg and EGC were isolated from hawthorn. A checkerboard method was used to determine which monomer or combination of the three monomers could produce the best ILSMR effect. The combination of C and EGC showed no ILSMR effect, with FICIs higher than 0.5 (Table S1). EGC in combination with ECg also had no ILSMR effect, with most FICIs above 0.5 and only one at 0.5 (Table S2). Significantly, the combination of C and ECg (Table 1) or of C, ECg and EGC showed strong ILSMR effects, with FICIs lower than 0.5 (Table 2), and there was no significant difference between two combinations. Because their pharmacological effects were equivalent, the more simple combination was preferentially chosen. Therefore, the combination of C and ECg was chosen and then its ILSMR effect was further investigated.

### 2.2. C and ECg Alone Have No ILSMR Effect, But When Combined, They Potentiate Antibacterial Effects of Oxacillin Against WHO-2

The MIC of C against WHO-2 was >1024 mg/L (no antibacterial effect), while that of ECg was 128 mg/L (very low antibacterial effect). C or ECg (32 mg/L) in combination with oxacillin produced FICIs of more than 0.5 or 0.5, demonstrating no or very weak ILSMR effect. Meanwhile, the FICI of C and ECg together with oxacillin was lower than 0.5, suggesting this combination had a strong ILSMR effect.

Two combinations of C and ECg at different concentrations, C (64 mg/L) + ECg (32 mg/L) or C (128 mg/L) + ECg (16 mg/L), combined with oxacillin produced FICIs lower than 0.375 (Table 1). Considering the good solubility of C and poor solubility of ECg in H_2_O, the latter combination with the lower ECg concentration, C (128 mg/L) + ECg (16 mg/L), was selected and investigated in the subsequent experiments.

### 2.3. C in Combination with ECg Specifically Potentiate the Antibacterial Effects of β-Lactam Antibiotics Against Clinical MRSA Strains

C in combination with ECg could potentiate the antibacterial effects of oxacillin against WHO-2. Therefore, it was of interest to determine whether C in combination with ECg could potentiate other β-lactam antibiotics or even non-β-lactam antibiotics and to verify the ILSMR effects, not only in WHO-2, but also in clinical MRSA strains. Herein, The 45 clinical MRSA strains were identified by drug susceptibility assay and amplification of the *mecA* gene. They all are resistant to oxacillin (MIC > 4 mg/L) and other five β-lactams with high levels of resistance. In the present experiment, the effects of C in combination with ECg to potentiate β-lactam antibiotics (oxacillin, ampicillin or ampicillin/sulbactam, cefazolin, cefepime and imipenem/cilastatin) and non-β-lactam antibiotics (vancomycin, linezolid and teicoplanin) against 45 clinical MRSA strains were observed.

The results showed that C or ECg each alone had no ILSMR effect, but C in combination with ECg could potentiate antibacterial effects of six β-lactam antibiotics (Table 3). Three combinations [C (32 mg/L) + ECg (4 mg/L), C (64 mg/L) + ECg (8 mg/L), and C (128 mg/L) + ECg (16 mg/L)] potentiated the effects of all six β-lactam antibiotics against almost all strains. However, none of these combinations could potentiate the effects of non-β-lactam antibiotics (Table S3).

### 2.4. C and ECg in Combination with Oxacillin Markedly Decrease Whole Blood Bacterial Load Than Oxacillin Alone in Mice Challenged with Sublethal WHO-2

**Figure 1:**
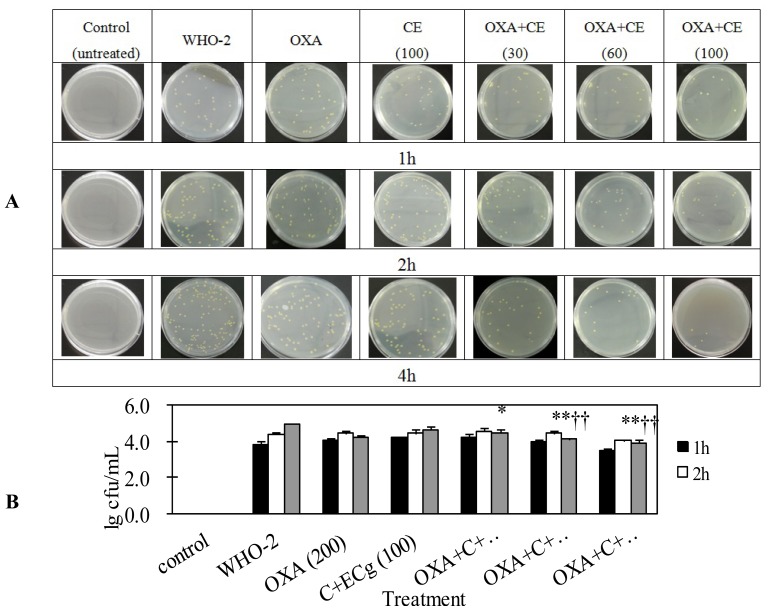
Effect of oxacillin (OXA) in combination with C and ECg on whole blood bacterial loads in mice treated with a sublethal dose of live MRSA. One hundred and five mice were randomly divided into seven groups (15 mice/group), and all were injected with live WHO-2 (4 × 10^9^ cfu/mL) except the control group (untreated). Four hours later, mice injected with bacteria were intragastrically injected with: (1) normal saline (NS) only; (2) oxacillin (200 mg/kg/day) only; (3) mixture of C and ECg (100 mg/kg/day) only; (4) mixture of C, ECg (30 mg/kg/day) and oxacillin (200 mg/kg/day); (5) mixture of C, ECg (60 mg/kg/day) and oxacillin (200 mg/kg/day); (6) mixture of C, ECg (100 mg/kg/day) and oxacillin (200 mg/kg/day). At indicated time points after the first treatment of C and ECg, 1 mL of blood from 3 mice of each group was collected at 1, 2 and 4 h time points after the first treatment of the agents, and samples were diluted with sterile normal saline immediately. The blood samples were then inoculated onto Müller**–**Hinton (MH) agar plates containing 50 mg/L of oxacillin. After culturing for 24 h at 37 °C, the bacterial colonies were counted and presented as lg cfu/mL of blood. (**A**) Photograph of MH agar plates containing bacterial colonies; (**B**) Statistical data of bacterial colonies presented as lg cfu/mL of blood. Data are presented as means ± standard deviation. * *p* < 0.05, compared to OXA group; ** *p* < 0.01, compared to OXA group; ^††^*p* < 0.01, compared to C and ECg mixture only group.

In order to determine whether C in combination with ECg could potentiate β-lactam antibiotics *in vivo*, the MRSA infection model was established in mice challenged with a sublethal dose of WHO-2. Considering the *t*_1/2_ of catechins was very short (approximately 1–4 h), blood bacterial loads were tested at 1, 2 and 4 h time points after the first treatment of C in combination with ECg [22]. The results showed there was no significant difference of bacterial blood load among mice treated with normal saline only, oxacillin only and C in combination with ECg. However, the bacterial load in mice treated with oxacillin (200 mg/kg/day) in combination with C and ECg was significantly lower than that in mice treated with oxacillin alone or C in combination with ECg (*p* < 0.05). The bacterial loads in mice treated with oxacillin in combination with C and ECg (100 mg/kg/day) was lowest (Figure 1). These results demonstrated C in combination with ECg potentiated oxacillin’s antibacterial effect *in vivo*, too.

### 2.5. C in Combination with ECg Increases Accumulation of Daunorubicin within WHO-2

The efflux system could confer multidrug resistance of MRSA. In order to investigate whether C and ECg demonstrated its ILSMR effect via increasing antibiotics accumulation, the influence of C and ECg on drug accumulation was investigated using fluorospectrophotometry assay and laser confocal scanning microscope observation.

**Figure 2:**
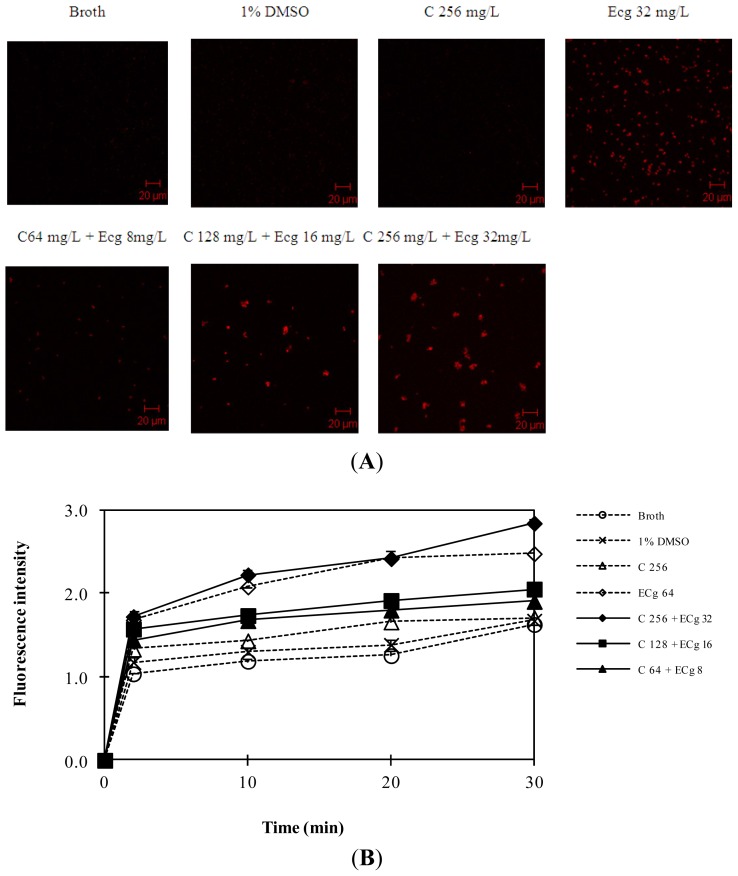
Daunorubicin accumulation within WHO-2 pretreated with C and ECg. WHO-2 was treated with broth, C (256 mg/L), ECg (32 mg/L) and C and ECg (64 mg/L + 8 mg/L, 128 mg/L + 16 mg/L, 256 mg/L + 32 mg/L) and then cultured for 6 h at 37 °C in a heated, shaking, environmental chamber. Then, the bacteria were centrifuged at 3500× *g* for 5 min. After washing three times and resuspending, the bacterial suspension was adjusted to an OD_600_ of 1.0. (**A**) Bacteria were incubated with daunorubicin (40 mg/L) in the dark at 37 °C for 30 min; 0.5 mL of bacteria was collected. Bacteria were washed three times and resuspended with PBS. Next, the bacteria were fixed on glass cover slips and observed under a 510 Meta confocal microscope (Zeiss, Göttingen, Germany); (**B**) Bacteria were incubated with daunorubicin (40 mg/L) in the dark at 37 °C for 2, 10, 20 and 30 min; 0.5 mL of bacteria was collected. Bacteria were washed three times and resuspended with phosphate buffered saline (PBS). Then, quantitative determination of daunorubicin accumulation in the absence and presence of C and ECg was performed using fluorospectrophotometry at an emission wavelength of 467 nm and an excitation wavelength of 588 nm.

Daunorubicin is not a β-lactam antibiotic but with red autofluorescence. It is used as a tracer agent because it can be observed more directly. The influence of daunorubicin was observed using drug susceptibility test and the result was shown by the time-kill curve (Figure S1). After it was confirmed that daunorubicin (20 and 40 mg/L) had no effect on the growth of WHO-2, the effect of C and ECg on the accumulation of daunorubicin within WHO-2 was detected. The results from confocal scanning microscopy showed C (256 mg/L) did not increase the daunorubicin accumulation within WHO-2 but ECg (32 mg/L) increased the drug accumulation. Significantly, C (256 mg/L) in combination with ECg (32 mg/L), increased more daunorubicin accumulation than ECg alone. Interestingly, the higher the concentration of C combined with ECg, the higher the daunorubicin accumulation (Figure 2A). The similar result was also observed from fluorospectrophotometry (Figure 2B). The above two results demonstrated that the ILSMR effect of C in combination with ECg was strongly related to the increased antibiotics’ accumulation.

### 2.6. C in Combination with ECg DownRegulates mRNA Expressions of Efflux Pumps within WHO-2

In order to investigate whether C in combination with ECg manifested its ILSMR effect via inhibiting mRNA expression of efflux pump genes, the influence of C in combination with ECg on mRNA expression of efflux pump genes were investigated using reverse transcription Polymerase Chain Reaction (RT-PCR) method.

**Figure 3:**
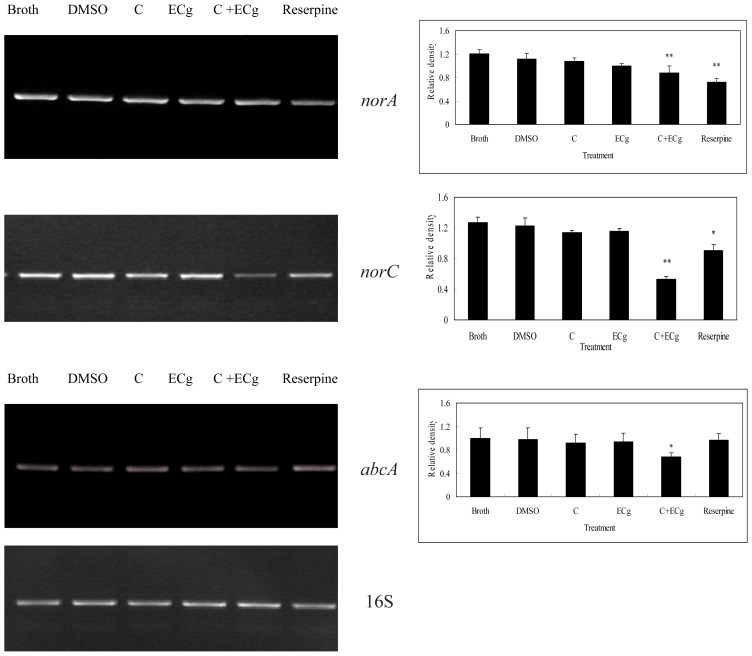
*norA*, *norC* and *abcA* mRNA expression within WHO-2. WHO-2 was treated with broth, 1% DMSO (*v*/*v*), C (128 mg/L), ECg (16 mg/L), C (128 mg/L) and ECg (16 mg/L) and Reserpine (20 mg/L), and cultivated at 37 °C and 100 g until an OD_600_ of 0.6 (6 h) was reached. After the bacterial harvest, total RNA was extracted and RT-PCR was performed. The signals for *norA*, *norC*, *abcA* and 16S rRNA were integrated on a Gel Doc 1000 Mini-Transilluminator (Bio-Rad, Hercules, CA, USA). 16S was used as a standard to allow semi-quantitative comparisons between samples. One-way analysis of variance (ANOVA) was used to examine the differences among different treatment (*n* = 3). * *p* < 0.05; ** *p* < 0.01 as compared with broth.

The results showed that reserpine as a positive control could downregulate mRNA expressions of *norA* and *norC* not *abcA*, which was in accordance with previous reports [23,24]. C or ECg alone did not influence efflux pump gene expression, but C in combination with ECg could downregulate mRNA expressions of *norA*, *norC* and *abcA* among eight efflux pumps of MRSA (Figure 3), thus suggesting that increased daunorubicin accumulation was related to the inhibition of three efflux pumps’ gene expressions.

### 2.7. Discussion

In traditional Chinese medicine, hawthorn is used as a peptic agent for stimulating digestion and promoting the function of the stomach, improving blood circulation and removing blood stasis. It is used as the agent for treatment of indigestion with epigastria distension, diarrhea, abdominal pain, amenorrhea, hypertension and hyperlipidemia, as well [25]. To the best of our knowledge, this is the first report demonstrating that hawthorn possesses ILSMR effects.

On the basis of the literature data, there are flavonoids, oligomeric proanthocyanidins, phenolicacids, triterpeneacids, organicacids and sterols within hawthorn fruit. Flavonoids are considered to be the main groups of active constituents in hawthorn extracts [21]. Catechins belong chemically to flavonoids.

Catechins are present in many plants and have many pharmacological effects. Although some of them may have the capacity to sensitize MRSA strains to oxacillin and other β-lactam antibiotics, they only possess weak antibacterial properties [26]. For example, galloy catechins, such as ECg and Cg, reduced the high MIC level of β-lactams to the antibiotic breakpoint or even lower than the breakpoint [14,15,26,27], but non-galloylated catechins, such as C and EC, had no such effect [15]. In the present study, we found ECg only had weak ILSMR effects, which did not accord with the previous reports [14,15], while C had no ILSMR effect as reported. The main reason could be that the ILSMR effect of ECg was influenced by its optical activity, which was affected by the extraction process and purity. Therefore, the discrepancies are likely due to different specific optical activities of ECg from the different sources of ECg and different MRSA strains.

We also found that when C combined with ECg, the ILSMR effect was markedly increased and the ILSMR effect of the combination (C and ECg) was enhanced with the increase in the concentration of C, with a higher concentration of C resulting in a lower FICI. This phenomenon did not occur when the concentration of ECg was increased, suggesting that the ILSMR effect of ECg could be potentiated by C. Previously, the cis form of non-galloylated catechins such as (−)-EC and (−)-EGC was reported to enhance the ILSMR effect of ECg [28], but excluding non-galloylated catechins of the trans form such as C. Herein, we firstly reported the ILSMR effects of C in combination with ECg on multiple classic β-lactam antibiotics against not only a standard MRSA strain but also clinical MRSA strains *in vitro* and *in vivo.*

In addition, C was found to be more effective than EC and EGC in potentiating the ILSMR effect of ECg against WHO-2 (data not shown). C and EC are known optical isomers. As the pharmacological effect of drugs requires strict chiral recognition by biological macromolecules [29], the bioactivities of optical isomers are generally different. The difference of chemical structure between C and EGC is that EGC has one more hydroxy group in the B ring than in that of C. Overall, the different abilities of C and EGC to potentiate the ILSMR effect of ECg was presumed to be due to the difference in steric hindrance provided by the hydroxy groups in the B ring of these compounds.

Previously, the ILSMR effect of ECg in combination with β-lactam antibiotics against the standard WHO-2 strain has been described [14,15]. Here, C together with ECg also showed an ILSMR effect on β-lactam antibiotics, not only against the MRSA WHO-2 standard strain, but also against 45 clinical MRSA strains, thereby suggesting the potential clinical value of this combination.

The ILSMR effect of ECg in combination with some β-lactam antibiotics against MRSA has also been described previously [14]. In this study, C in combination with ECg showed ILSMR effects on six β-lactam antibiotics: ampicillin, ampicillin/sulbactam, cefazolin, cefepime and imipenem/cilastatin. These β-lactam antibiotics are both classic and representative β-lactam antibiotics in the clinic. However, they cannot be used in the treatment of MRSA infections due to resistance. Our results showed that C in combination with ECg could significantly reduce the MIC of these six antibiotics against almost all of the clinical MRSA strains. These results are significant since they demonstrate the possibility that these antibiotics can be used in the clinic when combined with C and ECg. Interestingly, C in combination with ECg demonstrated ILSMR effects on β-lactam antibiotics, while they had no such effect on non-β-lactam antibiotics, which has not been reported previously.

Previously, ECg was found to prevent biofilm formation and promoted cell wall thickening, leading to cell aggregation without affecting the rate or extent of growth in culture [30]. Furthermore, ECg could insert into bacterial cytoplasmic membrane to disrupt PBP2a-mediated resistance by delocalizing PBP2 [31]. In the present study, we found that the ILSMR effect of C and ECg was also related to antibiotics accumulation. Efflux pumps were related to drug accumulation.

Efflux pumps were capable of extruding antibiotics, leading to multidrug resistance. There were nine efflux pumps within MRSA; they were *norA*, *norB*, *norC*, *mdeA*, *qacA/B*, *mepA*, *smr*, *sepA* [32], and *abcA* [33]. In a recent study, 50% of the 232 isolates of *S. aureus* were discovered to pump out at least two structurally unrelated substrates [34]. Frequencies of overexpressed efflux genes were *norA* (23%), *norB* (25%), *norC* (17%), *mdeA* (11%), and *mepA* (4%) [34], which meant *norA* and *norB* were the most popular pumps in *S. aureus*. However, *abcA* was reported to be responsible for the β-lactam resistance in *S. aureus* [33]. Previously, reserpine (a phytoalkaloid) was reported as efflux pump inhibitor (EPI) against various microbes [23,24]. There was one report that ECg inhibited *norA* efflux pump expression [35]. Herein, our results showed that reserpine down-regulated mRNA expressions of *norA* and *norC*, but not *abcA*, and C, in combination with ECg, down-regulated mRNA expressions of *norA*, *norC* and *abcA* among eight efflux pumps, while ECg or C alone could not. The above results suggested the ILSMR effect of C and ECg was probably related to the inhibition of three pumps’ mRNA expressions. Which pump(s) played a more important role should be further investigated in the future experiments.

In the present experiments, the synergy was assayed by FICI according to the criterion suitable for combination of only two compounds. Synergism was defined as an FICI ≤ 0.5 [36]. As is known, when more than two compounds were combined against bacteria, FICI would be easily more than 0.5, suggesting there was no synergism. In fact, each agent’s concentration had markedly decreased after combination. Therefore, a criterion suitable for more than two compounds should be investigated in the future.

## 3. Experimental Section

### 3.1. Materials

Chemicals used in extraction and isolation, like methanol, ethanol, ethyl acetate, normal butanol, acetone, were from Aladdin Regent Company (Shanghai, China). Macroporous D-101, silica gel and LH_20_ were from Ouyll Biological Technology Company (Tianjin, China). Oxacillin was purchased from Southwestern Pharmaceutical Corp. LTD. (Guangzhou, China). Ampicillin, cefazolin and cefepime were purchased from North China Pharmaceutical Group Corp. (Shijiazhuang, China). A 2:1 mixture of ampicillin sodium and sulbactam sodium was purchased from Seashore Pharmaceutical Company (Shenzhen, China). A 1:1 mixture of imipenem and cilastatin sodium was purchased from Zhuhai Federal Pharmaceutical Company (Zhuhai, China). Müller**-**Hinton (MH) agar plates and MH broth were purchased from OXOID Company (Basingstoke, UK).

### 3.2. Plant Material

The fruits of hawthorn (*Crataegus pinnatifida*) were collected from Yuzhou Market of Traditional Chinese Herbs, He Nan province in China, and identified by Yimin Zheng (Chongqing University of technology, Chongqing, China). The voucher specimen (2010-0900SZ^#^) of the hawthorn was deposited in our laboratory for future reference.

### 3.3. Bacterial Strains

The standard MRSA strain WHO-2 (WHO-2) and 45 clinical MRSA strains were kindly provided by Xiaoxing Luo [37] (The Fourth Military Medical University, Xi’an, China) and Peiyuan Xia (Southwestern Hospital, Chongqing, China), respectively. The properties of WHO-2 and 45 clinical MRSA strains were confirmed by both a drug susceptibility assay according to the Clinical Laboratory Standards Institute (CLSI) guidelines of 2010 (strains were resistant to oxacillin (MIC > 4 mg/L) and amplification of the *mecA* gene identified by Polymerase Chain Reaction (PCR) in our lab. WHO-2 possessed a high level of resistance to oxacillin (MIC was 512 mg/L) and harbored the *mecA* gene. Forty-five clinical strains were all resistant to oxacillin (MIC > 4 mg/L) and harbored the *mecA* gene. The primers of *mecA* gene and 16S were listed (Table S4).

### 3.4. Animals

Specific-Pathogen-Free (SPF) Kunming (KM) mice (4–6 weeks old, weighing 18–22 g) were supplied by the Experimental Animal Center of the Third Military Medical University (Chongqing, China). An equal number of male and female mice were used. All animals received humane care in compliance with the Principles of Laboratory Animal Care formulated by the National Society for Medical and Research and the “Guide for the Care and Use of Laboratory Animals” prepared by the National Academy of Sciences (Washington DC, USA). The experiments were approved by a medical ethics committee of The Third Military Medical University.

### 3.5. Extraction and Isolation

**Figure 4:**
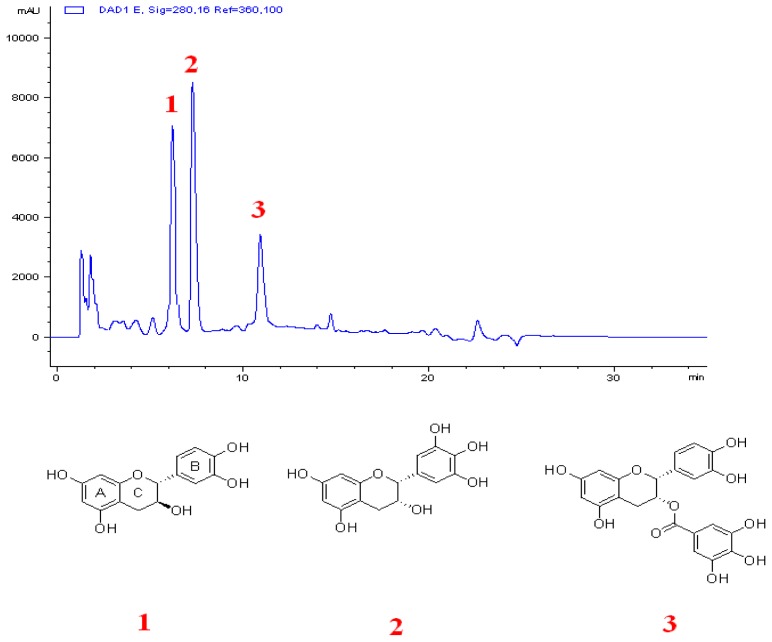
The figure showed the HPLC-DAD chromatogram of the effective fraction (fraction 4) which subjected to the silica gel column, and the 3 active compounds were isolated from fraction 4. An Agilent SB C-18 reversed-phase column (150 × 4.6, 5 μm) was used with column temperature set at 30 °C, and the spectrum at absorbance of 280 nm. The mobile phase consisted of methanol (20%) and 0.5% aqueous glacial acetic acid (80%), and the flow rate was 1 mL/min. 1–3 represented (+)-catechin, (−)-epicatechin gallate and (−)-epigallatecatechin, respectively.

The fruits of hawthorn were crushed and refluxing extracted with 75% EtOH three times (each extraction period lasted 2 h). Merging the extraction together, the 75% EtOH extract was portioned between EtOAc, n-BuOH. Each extract (EtOAc extract, n-BuOH extract, H_2_O extract) was measured for antibacterial activity and ILSMR effect. The active part was chromatographed on macroporous D-101 column (washed by EtOH-H_2_O), and 50% EtOH-H_2_O fraction was collected by the freeze-drying technique. The sample was chromatographed on a silica gel column with chloroform and acetone afforded the fraction1–8 (frs.1–8), and then these fractions were measured for antibacterial effect and ILSMR effect. Fraction 4, which showed a significant ILSMR effect, was then separated by Sephadex LH-20 column chromatography and preparative HPLC (Agilent 1100 Series equipped with G1361A binary pump, G1365B MWD UV detector, G1364B fraction collector), yielding the three monomeric compounds. The three compounds were identified as (+)-catechin, (−)-epicatechin gallate and (−)-epigallatecatechin by the methods of ^1^H-NMR and spiking with reference compounds which were purchased or available in our laboratory (Figure 4).

### 3.6. Bacterial Growth

Each single colony from the MH agar plate was inoculated into a 10 mL volume of liquid MH broth (containing 2% NaCl), according to the CLSI 2010 guidelines, and cultivated aerobically at 37 °C in a heated and shaking chamber for 12 h. These cultures were diluted 1:100 (*v*/*v*) in another 10 mL of fresh broth and cultivated aerobically until the log growth phase.

### 3.7. Drug Susceptibility Assay

Bacteria in exponential phase (1 × 10^5^ cfu/mL) were inoculated into 96-well plates. MIC of β-lactam antibiotics, C alone and ECg alone were determined by serial two-fold dilutions in MH broth according to CLSI 2010 guidelines. MIC_50_ and MIC_90_ were the half and 90% MICs of β-lactam antibiotics against 45 clinical MRSA strains, respectively.

Synergistic effects were assayed by determining the FICI calculated for each combination using the following formula: FICI = (MIC of combined antibiotic/MIC of antibiotic alone) + (concentration of combined “C”/MIC of “C” alone) + (concentration of combined “ECg”/MIC of “ECg” alone). Synergism was defined as a FICI ≤ 0.5, antagonism was defined as an FICI > 4.0, and no interaction was defined with a FICI 0.5–4.0, according to previously described criteria [36].

### 3.8. Experimental Animal Model and Drug Treatment

One hundred and five mice, weighing from 18 g to 22 g, were randomly divided into seven groups (15 mice/group) and intravenously injected with a sublethal dose of live WHO-2 (4.0 × 10^9^ cfu/mL). Four hours later, the mice were given the following treatments (C and ECg mixture by intragastric injection and oxacillin by intramuscular injection): Normal saline only, oxacillin (200 mg/kg/day) only; mixture of C and ECg (total dose 100 mg/kg/day); combination of C and ECg mixture (100 mg/kg/day) with oxacillin (200 mg/kg/day); combination of C and ECg mixture (60 mg/kg/day) and oxacillin (200 mg/kg/day); combination of C and ECg mixture (30 mg/kg/day) and oxacillin (200 mg/kg/day). The total injection volume was 0.4 mL/20 g body weight. One milliliter of blood from each of three mice of each group was collected at 1-, 2- and 4-h time points after the first treatment of the agents, and samples were diluted with sterile normal saline immediately. The blood samples were then inoculated onto MH agar plates containing 50 mg/L of oxacillin. After culturing for 24 h at 37 °C, the bacterial colonies were counted and presented as lg cfu/mL of blood.

The dose of oxacillin used in this study was based on a conversion of clinical dosage regimens. Doses of oxacillin in the range from 30–100 mg/kg/day have been proposed to treat bacterial infection in adult patients, and higher doses are given for serious infections. According to the drug conversion principle applied in pharmacological studies, a dose ranging from 30–100 mg/kg/day of oxacillin in adults is equivalent to that of 300–900 mg/kg/day in mice. Therefore, the dose of 200 mg/kg/day of oxacillin chosen for mice in this study was lower than the equivalent doses used for humans in the clinic.

C and ECg were combined at the mass ratio of 8:1 and then mixed with sodium carboxymethylcellulose (0.5%) by milling. The dose of the C and ECg mixture used in mice was based on results of *in vitro* experiments, which showed that the doses of C at 128 mg/L and ECg at 16 mg/L (144 mg/L total) resulted in the best ILSMR effect. Because the quantity of body fluid in a mouse is generally 70%–80% of its body weight (average 20 g per mouse), the dose of the C and ECg mixture should be about 2 mg per mouse. Therefore, the highest dose of the C and ECg mixture used was calculated at 100 mg/kg/day in mice.

### 3.9. Accumulation of Daunorubicin within WHO-2

WHO-2 was inoculated into 10 mL of MH broth with seven different treatments: Broth only, 1% (*v*/*v*) dimethyl sulfoxide (DMSO), C (256 mg/L), ECg (64 mg/L), the combination of C (64 mg/L) and ECg (8 mg/L), the combination of C (128 mg/L) and ECg (16 mg/L), and the combination of C (256 mg/L) and ECg (64 mg/L). Then the mixtures were cultured for 6 h at 37 °C in a heated and shaking chamber. Then, the bacteria were centrifuged at 3500× *g* for 5 min to harvest the bacterial pellet. After washing with PBS (0.2 mM, pH 7.2) three times, the bacterial pellet was resuspended and the bacterial suspension was adjusted to an OD_600_ of 1.0. After co-culture with daunorubicin (40 mg/L) at 37 °C for 2, 10, 20 and 30 min in the dark, daunorubicin accumulation within WHO-2 was observed by the two methods described below.

The first method was fluorospectrophotometry; daunorubicin accumulation within the bacteria was assayed in the absence or presence of C and ECg. The emission wavelength was 467 nm and the excitation wavelength was 588 nm. Additionally, as the second method, at 30 min, 0.5 mL of the bacteria was collected. After washing with PBS three times, the bacteria were fixed on glass cover slips and observed under a 510 Meta confocal microscope (Zeiss, Göttingen, Germany).

### 3.10. mRNA Expression Assays

WHO-2 was inoculated into 100 mL of MH broth with six different treatments: broth, 1% DMSO (*v*/*v*), C (128 mg/L), ECg (16 mg/L), C (128 mg/L) and ECg (16 mg/L) and reserpine (20 mg/L). Bacteria were cultivated to an OD_600_ of 0.6 and washed with normal saline. Then bacteria were harvested and milled with liquid nitrogen. 1 mL of RNAiso was added to completely lyse bacteria. Chloroform was added to the mixture with shaking for 15 s and stewing for 10 min. Bacteria were harvested by centrifugation at 10,000× *g* for 15 min in 4 °C. Isopropanol was added with stewing for 15 min. Bacteria were harvested again by centrifugation at 12,000× *g* for 10 min and washed by 75% (*v*/*v*) ethanol, and then 20 μL of RNase-free ddH_2_O was added. The reverse transcription was performed by PrimeScript RT reagent Kit. The primers for *mecA*, *norA*, *norB*, *norC*, *mepA*, *mdeA*, *sepA*, *qacA/B*, *abcA*, *smr* and 16S RNA were listed (Table S1). The primers were added to the PCR tubes and subjected to the normal reverse transcription PCR (RT-PCR). RT-PCR products were determined using 1.0% agarose gel electrophoresis and Golden View staining. Images of the gels were analyzed using Quantity One software (Bio-Rad, Hercules, CA, USA).

### 3.11. Statistical Analysis and Presentation of Data

Each experiment was repeated at least three times, and each data point was represented as the mean, with error bars denoting standard deviations. Differences in bacterial load in the whole blood of mice were examined by repeated measures ANOVA using Statistical Program for Social Sciences (SPSS) 16.0. A *p* value of 0.05 was considered significant, and a *p* value of 0.01 was considered highly significant.

## 4. Conclusions

In conclusion, C in combination with ECg enhanced the antibacterial effect of β-lactam antibiotics against MRSA *in vitro* and *in vivo*, which might be related to the increased antibiotics accumulation within MRSA via suppression of the important efflux pumps’ gene expression.

## Supplementary Information



## Figures and Tables

**Table 1:** Minimum inhibitory concentrations (MIC) of oxacillin in combination with C and ECg against WHO-2. Synergistic activities of different concentrations of C, ECg and oxacillin (OXA) on WHO-2 were tested by the checkerboard method. Fractional inhibitory concentration index (FICI) values were calculated as described in Experimental Section.

C (mg/L) a	ECg (mg/L) b	MIC of OXA (mg/L)	FICI
0	0	512	cannot calculate
	8	512	1.625
	16	256	0.625
	32	128	0.500

16	0	512	<1.016
	8	512	<1.073
	16	256	<0 636
	32	64	<0.386

32	0	512	<1.031
	8	512	<1.094
	16	256	<0.656
	32	64	<0.406

64	0	512	<1.063
	8	256	<0.625
	16	128	<0.438
	32	32	<0.375

128	0	512	<1.125
	8	128	<0.438
	16	64	<0.375
	32	<4	<0.383

256	0	512	<1.250
	8	64	<0.438
	16	16	<0.406
	32	<4	<0.508

512	0	256	<1.000
	8	16	<0.531
	16	<4	<0.633
	32	<4	<0.758

a For C, the MIC against WHO-2 was >1024 mg/L;

b For ECg, the MIC against WHO-2 was 128 mg/L.

**Table 2:** MIC of oxacillin in combination with C, ECg and EGC against WHO-2. Testing of synergy among C, EGC and oxacillin (OXA) was performed using the checkerboard method. FICI values were calculated as described in Experimental Section.

C a	EGC b	ECg c	OXA d MIC (mg/L)	FICI
16	16	8	256	<0.641
		16	128	<0.453
		32	32	<0.391

	32	8	256	<0.703
		16	128	<0.516
		32	32	<0.454

	64	8	256	<0.829
		16	128	<0.641
		32	<4	<0.524

32	16	8	256	<0.656
		16	128	<0.469
		32	16	<0.375

	32	8	256	<0.719
		16	128	<0.531
		32	16	<0.438

	64	8	128	<0.594
		16	64	<0.531
		32	<4	<0.535

64	16	8	128	<0.438
		16	128	<0.475
		32	16	<0.375

	32	8	128	<0.469
		16	64	<0.406
		32	<4	<0.409

	64	8	128	<0.594
		16	32	<0.468
		32	<4	<0.534

128	16	8	128	<0.500
		16	32	<0.438
		32	<4	<0.441

	32	8	128	<0.375
		16	32	<0.438
		32	<4	<0.503

	64	8	16	<0.469
		16	<4	<0.316
		32	<4	<0.628

256	16	8	64	<0.500
		16	16	<0.469
		32	<4	<0.568

	32	8	64	<0.563
		16	32	<0.563
		32	16	<0.656

	64	8	<4	<0.565
		16	<4	<0.628
		32	<4	<0.753

512	16	8	32	<0.688
		16	<4	<0.671
		32	<4	<0.815

	32	8	16	<0.719
		16	<4	<0.754
		32	<4	<0.878

	64	8	<4	<0.815
		16	<4	<0.878
		32	<4	<1.003

a For C, the MIC against WHO-2 was >1024 mg/L;

b For EGC, the MIC against WHO-2 was 256 mg/L;

c For ECg, the MIC against WHO-2 was 128 mg/L;

d For OXA, the MIC against WHO-2 was 512 mg/L.

**Table 3:** Effect of C and ECg on antibacterial effects of six β-lactam antibiotics against 45 clinical MRSA strains. Synergistic activities of C and ECg in combination with six β-lactams antibiotics (oxacillin, ampicillin, ampicillin/sulbactam, cefazolin, cefepime and imipenem/cilastatin) were tested by checkerboard method. FICI values were calculated as described in Experimental Section.

Antibiotics	MIC (mg/L) of β-lactam antibiotics										
	Concentration of C a + ECg b (mg/L)										

	0			16 + 2				32 + 4			
		
	Range	MIC_50_	MIC_90_	Range	MIC_50_	MIC_90_	FICI c	Range	MIC_50_	MIC_90_	FICI c
oxacillin	128–512	256	512	4–512	128	256	0.56 ± 0.32 (13/45)	4–256	32	256	0.31 ± 0.22 (36/45)
ampicillin	32–512	64	128	2–256	16	64	0.37 ± 0.26 (30/45)	2–256	8	32	0.29 ± 0.24 (39/45)
ampicillin/sulbactam	8–32	32	32	2–32	16	32	0.58 ± 0.29 (14/45)	2–16	8	16	0.39 ± 0.23 (33/45)
cefazolin	4–512	256	512	4–512	128	256	0.47 ± 0.33 (20/45)	4–256	32	128	0.25 ± 0.18 (39/45)
cefepime	8–2048	1024	1024	8–1024	256	512	0.45 ± 0.23 (18/45)	8–1024	128	512	0.30 ± 0.20 (39/45)
imipenem/cilastatin	4–512	128	256	4–512	32	64	0.36 ± 0.25 (29/45)	4–512	4	32	0.23 ± 0.25 (41/45)

**Antibiotics**		**MIC (mg/L) of β-lactam antibiotics**									
		**Concentration of C**^a^**+ ECg**^b^**(mg/L)**									

		**64 + 8**						**128 + 16**			
	
		**Range**		**MIC_50_**	**MIC_90_**	**FICI**^c^		**Range**	**MIC_50_**	**MIC_90_**	**FICI**^c^

oxacillin		4–256		4	128	0.28 ± 0.19 (42/45)		4–256	4	4	0.41 ± 0.22 (31/45)
ampicillin		2–256		2	16	0.27 ± 0.19 (42/45)		2–256	2	2	0.29 ± 0.22 (31/45)
ampicillin/sulbactam		2–16		2	4	0.30 ± 0.19 (42/45)		2–16	2	2	0.44 ± 0.12 (33/45)
cefazolin		4–64		4	32	0.23 ± 0.15 (44/45)		4–4	4	4	0.39 ± 0.21 (32/45)
cefepime		8–256		8	64	0.25 ± 0.17 (44/45)		8–16	8	8	0.39 ± 0.23 (32/45)
imipenem/cilastatin		4–512		4	4	0.28 ± 0.30 (42/45)		4–512	4	4	0.45 ± 0.26 (32/45)

a For C, the MIC range, MIC_50_, MIC_90_ against 45 MRSA clinical strains were 512–4096 mg/L, 2048 mg/L and 4096 mg/L, respectively;

b For ECg, the MIC range, MIC_50_, MIC_90_ against 45 MRSA clinical strains were 32–1024 mg/L, 64 mg/L and 1024 mg/L, respectively;

c Data are expressed as means ± standard deviations. Values in parentheses represent the number of strains for which drug combinations resulted in synergism/number of strains tested.
