# Supplementary material for: The Combination of Catechin and Epicatechin Gallate from Fructus Crataegi Potentiates β-Lactam Antibiotics Against Methicillin-Resistant Staphylococcus aureus (MRSA) in Vitro and in Vivo
Source: Int J Mol Sci. 2013 Jan 16;14(1):1802–21. doi: 10.3390/ijms14011802 (PMC3565349; doi:10.3390/ijms14011802)
Supplement: Supplementary file 1 [file ijms-14-01802-s001.pdf]

Supplementary Materials

Table S1. Synergy of oxacillin in combination with C and EGC against WHO-2. Testing of synergy between different concentrations of C/EGC and oxacillin (OXA) was performed using the checkerboard method. FICI values were calculated as described in Experimental Section. [a] For C, the MIC of C against WHO-2 was >1024 mg/L; [b] For EGC, the MIC against WHO-2 was 256 mg/L.

**C** [a]	**EGC** [b]	**OXA MIC (mg/L)**	FICI
0	0	512	cannot calculate
	16	512	1.063
	32	512	1.125
	64	512	1.250
16	0	512	<1.016
	16	512	<1.078
	32	512	<1.141
	64	512	<1.266
32	0	512	<1.031
	16	512	<1.095
	32	512	<1.157
	64	512	<1.282
64	0	512	<1.063
	16	256	<0.626
	32	256	<0.688
	64	256	<0.578
128	0	512	<1.125
	16	512	<1.188
	32	512	<1.250
	64	256	<0.750
256	0	512	<1.250
	16	512	<1.313
	32	512	<1.375
	64	256	<1.000
512	0	256	<1.000
	16	128	<0.813
	32	32	<0.688
	64	<4	<0.750

Table S2. Synergy of oxacillin in combination with EGC and ECg against WHO-2. Testing of synergy between EGC, ECg and oxacillin (OXA) at different concentrations in the inhibition of WHO-2 was performed using the checkerboard method. FICI values were calculated as described in Experimental Section. [a] For EGC, the MIC against WHO-2 was 256 mg/L; [b] For ECg, the MIC against WHO-2 was 128 mg/L.

EGC [a]	ECg [b]	MIC of OXA (mg/L)	FICI
0	0	512	cannot calculate
	8	512	1.625
	16	256	0.625
	32	128	0.500
16	0	512	1.063
	8	512	1.125
	16	512	1.187
	32	256	0.813
32	0	512	1.125
	8	256	0.688
	16	256	0.750
	32	128	0.563
64	0	512	1.250
	8	256	0.563
	16	64	0.500
	32	64	0.625
128	0	512	1.5
	8	32	0.625
	16	<4	<0.625
	32	<4	<0.750

Table S3. FICI values of C and ECg in combination with non-β-lactam antibiotics against MRSA WHO-2. Synergistic activities of different concentrations of C, ECg and anti-MRSA antibiotics including vancomycin, linezolid and teicoplanin were tested against 22 clinical MRSA strains by checkerboard method. FICI values were calculated as described in Experimental Section. [a] The MIC range, MIC_{50}, MIC_{90} against 45 clinical MRSA strains were 512–4096 mg/L, 2048 mg/L and 4096 mg/L for C, respectively; [b] and 32–1024 mg/L, 64 mg/L and 1024 mg/L for ECg, respectively; [c] Data were expressed as means ± standard deviations. Values in parentheses indicated the number of strains for which drug combinations resulted in synergism/number of strains tested.

Antibiotics	MIC of non-β-lactam antibiotics (mg/L) concentration of C [a] + concentration of ECg [b] (mg/L)														
	0			32 + 4				64 + 8				128 + 16			
	Range	MIC_{50}	MIC_{90}	Range	MIC_{50}	MIC_{90}	FICI [c]	Range	MIC_{50}	MIC_{90}	FICI [c]	Range	MIC_{50}	MIC_{90}	FICI [c]
vancomycin	2−64	4	8	2−64	4	8	1.30 ± 0.61 (2/22)	2−64	4	8	1.34 ± 0.69 (2/22)	0.25−64	0.25	8	0.83 ± 0.60 (8/22)
linezolid	0.5−64	2	4	0.25−64	8	8	1.04 ± 0.53 (2/22)	0.25−64	1	8	1.15 ± 0.58 (2/22)	0.25−64	0.5	4	0.95 ± 0.57 (8/22)
teicoplanin	1−64	4	16	0.25−64	2	8	1.16 ± 0.66 (4/ 2)	0.25−8	8	8	1.15 ± 0.89 (4/22)	0.25−64	8	8	0.88 ± 0.58 (3/22)

Table S4. The primers for *mecA*, *norA*, *norB*, *norC*, *mepA*, *mdeA*, *sepA*, *qacA/B*, *abcA*, *smr* and 16S RNA.

Gene		Primer	Length of product
mecA	PF	5′-GATTATGGCTCAGGTACTGCTATCC-3′	
	PR	5′-ATGAAGGTGTGCTTACAAGTGCTAA-3′	70 bp
norA	PF	5′-CGGTCTAGTGATACCAGTCT-3′	
	PR	5′-AACCATACCAGCACTCATAC-3′	268 bp
norB	PF	5′-AGCGCGTTGTCTATCTTTCC-3′	
	PR	5′-GCAGGTGGTCTTGCTGATAA-3′	213 bp
norC	PF	5′-AATGGGTTCTAAGCGACCAA-3′	
	PR	5′-ATACCTGAAGCAACGCCAAC-3′	216 bp
mepA	PF	5′-TGCTGCTGCTCTGTTCTTTA-3′	
	PR	5′-GCGAAGTTTCCATAATGTGC-3′	198 bp
medA	PF	5′-GTTTATGCGATTCGAATGGTTGGT-3′	
	PR	5′-AATTAATGCAGCTGTTCCGATAGA-3′	155 bp
sepA	PF	5′-GCAGTCGAGCATTTAATGGA-3′	
	PR	5′-ACGTTGTTGCAACTGTGTAAGA-3′	103 bp
smr	PF	5′-ATTGGAAGTGCATTTCTTAA-3′	
	PR	5′-AACGAAACTACGCCGACTAT-3′	257 bp
qacA/B	PF	5′-TGGCTTCACTAGCAGTTGCA-3′	
	PR	5′-ACAGCGCCCACTACAGATTC-3′	238 bp
abcA	PF	5′-GTCGGTGCAAGTAGTAGAAT-3′	
	PR	5′-TTTACCAGACCCAGAAGG-3′	216 bp
16S	PF	5′-GAGAGAAGGTGGGGATGACGT-3′	
	PR	5′-AGGCCCGGGAACGTATTCAC-3′	217 bp

Figure S1. The influence of daunorubicin on the growth of MRSA WHO-2. Each single colony from the MH agar plate was inoculated into a 10 mL volume of MH broth (containing 2% NaCl) according to the CLSI 2010 guidelines and cultivated aerobically at 37 °C in a heated and shaking chamber for 12 h to reach OD600 = 0.6. These cultures were treated with different concentrations of daunorubicin of 0 mg/L, 20 mg/L and 40 mg/L and cultivated in the dark at 37 °C, 100 g for 0.5, 1.5 and 2 h. 100 μL of bacteria was collected and measured at OD600. Result was shown with time-kill curve.

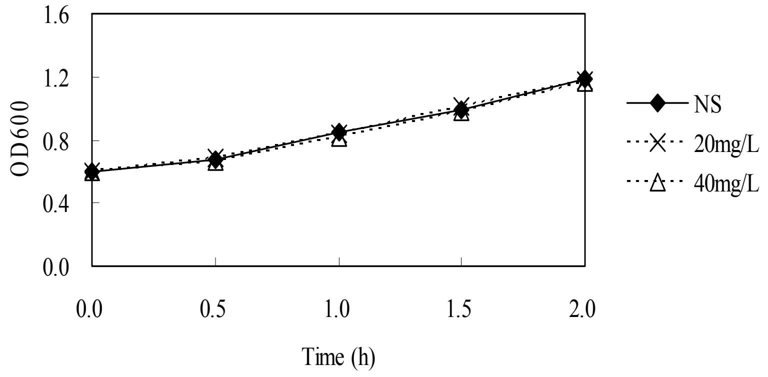

© 2013 by the authors; licensee MDPI, Basel, Switzerland. This article is an open access article distributed under the terms and conditions of the Creative Commons Attribution license (http://creativecommons.org/licenses/by/3.0/).
